# Screen Time Weight-loss Intervention Targeting Children at Home (SWITCH): process evaluation of a randomised controlled trial intervention

**DOI:** 10.1186/s12889-016-3124-8

**Published:** 2016-05-26

**Authors:** Louise Foley, Cliona Ni Mhurchu, Samantha Marsh, Leonard H. Epstein, Tim Olds, Ofa Dewes, Ihirangi Heke, Yannan Jiang, Ralph Maddison

**Affiliations:** MRC Epidemiology Unit and UKCRC Centre for Diet and Activity Research (CEDAR), University of Cambridge School of Clinical Medicine, Box 285, Cambridge Biomedical Campus, Cambridge, CB2 0QQ UK; National Institute for Health Innovation, School of Population Health, University of Auckland, Private Bag 92019, Auckland Mail Centre, Auckland, 1142 New Zealand; Departments of Pediatrics, Community Health and Health Behavior and Social and Preventive Medicine, University at Buffalo School of Medicine and Biomedical Sciences, 3435 Main Street, G56 Farber Hall, Buffalo, NY 14214 USA; Alliance for Research in Exercise, Nutrition and Activity (ARENA), University of South Australia, City East Campus, Frome Road, GPO Box 2471, Adelaide, SA 5001 Australia; Pacific Health, School of Population Health, University of Auckland, Private Bag 92019, Auckland Mail Centre, Auckland, 1142 New Zealand; Heke Consulting, Auckland, 1142 New Zealand

## Abstract

**Background:**

The Screen Time Weight-loss Intervention Targeting Children at Home (SWITCH) trial tested a family intervention to reduce screen-based sedentary behaviour in overweight children. The trial found no significant effect of the intervention on children’s screen-based sedentary behaviour. To explore these null findings, we conducted a pre-planned process evaluation, focussing on intervention delivery and uptake.

**Methods:**

SWITCH was a randomised controlled trial of a 6-month family intervention to reduce screen time in overweight children aged 9–12 years (*n* = 251). Community workers met with each child’s primary caregiver to deliver the intervention content. Community workers underwent standard training and were monitored once by a member of the research team to assess intervention delivery. The primary caregiver implemented the intervention with their child, and self-reported intervention use at 3 and 6 months. An exploratory analysis determined whether child outcomes at 6 months varied by primary caregiver use of the intervention.

**Results:**

Monitoring indicated that community workers delivered all core intervention components to primary caregivers. However, two thirds of primary caregivers reported using any intervention component “sometimes” or less frequently at both time points, suggesting that intervention uptake was poor. Additionally, analyses indicated no effect of primary caregiver intervention use on child outcomes at 6 months, suggesting the intervention itself lacked efficacy.

**Conclusions:**

Poor uptake, and the efficacy of the intervention itself, may have played a role in the null findings of the SWITCH trial on health behaviour and body composition.

**Trial registration:**

The trial was registered in the Australian and New Zealand Clinical Trials Registry (no. ACTRN12611000164998); registration date: 10/02/2011.

## Background

Public health interventions with a goal of improving health-related behaviour (e.g., physical activity or sedentary behaviour) are often deemed complex in that they have multiple interacting components, implementation is usually not fully standardised across participants, and it is difficult to separate the intervention from the context in which it operates [1].

A trial can identify *if* a complex public health intervention worked; however, a process evaluation is recommended to understand *how* it worked (or failed to work) [1, 2] through examination of the intervention content, implementation and context. Whether or not statistically or clinically significant changes in target behaviours or health outcomes are observed, a process evaluation can give insight into the intervention’s “active ingredients”. In particular, a process evaluation may be useful to distinguish between a non-efficacious intervention and one that has failed for other reasons (e.g., poor implementation) [3]. Even subtle variations in implementation, participants and context may explain why the effects of similar interventions vary – sometimes markedly - across studies [4].

The Screen Time Weight-loss Intervention Targeting Children at Home (SWITCH) trial, based in Auckland, New Zealand (NZ), was a 6-month family intervention to reduce screen-based sedentary behaviour in overweight children aged 9–12 years. The intervention was distinct from a previous public health intervention by the same name [5]. The SWITCH intervention was grounded in Social Cognitive Theory and Behavioural Economics Theory, and the trial was conducted between 2010 and 2012. The methods – including a detailed description of the underpinning evidence and theory-based intervention [6] - and main results [7] of this study have been reported. The SWITCH study found no significant effect of the intervention on children’s screen-based sedentary behaviour (−33.2 min/day, 95 % CI −73.3 to 7.0, *p* = 0.11) or body mass index z-score (zBMI) (−0.02 units, 95 % CI −0.08 to 0.05, *p* = 0.64) at 6 months [7].

Why was the SWITCH intervention unsuccessful, and what lessons may be applied to refining future interventions? While myriad factors may have contributed, we chose to narrow our focus to intervention implementation, an element essential to intervention success and one which could feasibly be assessed considering both budgetary constraints and participant burden. Implementation is a critical factor in pragmatic trials where interventions are often flexibly applied, and particularly in this trial where the intervention was not directly delivered to children by the study team. This approach is consistent with current recommendations to focus on the most important questions when conducting a process evaluation [2]. The overall aim of the study was to conduct a pre-planned process evaluation of the SWITCH trial. The specific objectives were to (a) describe the delivery and uptake of specific intervention elements, and (b) conduct an exploratory analysis to determine whether level of uptake of the intervention influenced child activity or body composition outcomes.

## Methods

The methods of the SWITCH trial, including a sample size calculation and definition of the primary and secondary trial outcomes, have been reported [6, 7] and are briefly described below.

### Design

The SWITCH trial was a two-arm, parallel, randomised controlled trial of a 6-month family intervention to reduce screen-based sedentary behaviour in overweight children aged 9–12 years (*n* = 251). Ethical approval was received from the Lower South Regional Ethics Committee (LSR/10/09/039) and the trial was registered (ACTRN12611000164998). Written informed consent was provided by primary caregivers and written assent by children.

### Procedure

Participants were recruited into the trial via schools, community centres and word of mouth. Following baseline assessment, participants and their primary caregiver were randomised at a 1:1 ratio to intervention (*n* = 127) or control (*n* = 124) via centralised computer randomisation, using stratified blocked randomisation by sex and ethnicity using variable block sizes, and maintaining allocation concealment in order to minimise the risk of allocation or selection biases. Intervention primary caregivers received a single one-hour face-to-face meeting, in which a SWITCH community worker discussed strategies for reducing their child’s screen time, fitted a Time Machine television budgeting device, and provided an activity pack containing apparatus for alternative activities (e.g., a ball and colouring pencils). Following this meeting, primary caregivers received five monthly newsletters outlining additional strategies for reducing their child’s screen time. Control participants continued with usual activities and were given the intervention materials at the end of the study. There was no blinding in this pragmatic trial.

### Assessment

At baseline and 6 months, assessments were undertaken at the participant’s home. The following were assessed: measured height and weight (child and primary caregiver); measured body composition using bioelectrical impendence (child); self-reported physical activity and sedentary behaviour using the Multimedia Activity Recall for Children and Adolescents [8] (child) and the International Physical Activity Questionnaire long form [9] (primary caregiver); self-reported perceived enjoyment of physical activity and sedentary behaviour (child) and self-reported dietary intake using a semi-quantitative food frequency questionnaire [10] (child). An analysis of covariance (ANCOVA) regression model was used to evaluate the main trial treatment effect on change from baseline for all variables, adjusting for baseline value, age, sex, and ethnicity, using an intention-to-treat approach. The primary outcome was change from baseline in child BMI (kg/m^2^) and zBMI [7].

Additional detail is provided on the assessment of intervention delivery and use, which were examined in detail in the process evaluation. While the intervention was grounded in Social Cognitive Theory and Behavioural Economics Theory, the process evaluation itself was not explicitly theory- or framework- driven.

### Intervention delivery

SWITCH community workers underwent standard training (approximately three hours) with a member of the investigative team (LF).

Community workers were introduced to the theoretical underpinning of the intervention and trained to deliver the following core intervention components to primary caregivers at the face-to-face meeting (see protocol [6] for a more detailed description):

- Define screen time and give examples
- Screen time reduction strategy 1: Praise
- Screen time reduction strategy 2: Positive reinforcement
- Screen time reduction strategy 3: Environmental control
- Screen time reduction strategy 4: Budgeting and self-monitoring (Time Machine device)
- Screen time reduction strategy 5: Alternate activities (activity pack)

Community workers were trained to deliver the intervention material to primary caregivers in a broadly standardised way, with the understanding that they were still one step removed from the intervention target (the child), and therefore unable to directly control the implementation of the intervention. This “train-the-trainers” model has been used successfully in national programs in NZ [11]. A core thread of the training for the face-to-face meeting involved emphasising the primary caregiver’s identity as a positive family role model, and their ultimate autonomy to implement the intervention as they saw fit.

Community workers were trained in motivational interviewing techniques to draw out the primary caregiver’s motivation to change and work towards a solution the primary caregiver felt was feasible. Additionally, IH conducted further training emphasising the NZ cultural context. IH also taught community workers several culturally-appropriate games that could be introduced to families as an alternative to screen time.

Following training and the initiation of fieldwork, community workers were monitored once by LF during a face-to-face meeting with a primary caregiver. With permission from the primary caregiver, LF attended the meeting as an observer. LF completed a standard checklist to identify whether core intervention components were delivered, as well as writing notes on the general conduct and success of the meeting. The monitoring took place within the first six months of the initiation of fieldwork, and the aim was to ensure that the core intervention components were being delivered. A monitoring report was produced, which gave feedback and recommendations for the community worker to implement in future meetings. Given that community workers were only monitored once, the monitoring was not intended or able to document intervention delivery across all 127 intervention families, but rather gave an indication of the consistency with which the intervention was delivered by different community workers.

### Intervention use/uptake

Intervention primary caregiver’s self-reported use of the intervention strategies was assessed at 3 months via a phone call, and 6 months at the final assessment in the participant’s home.

At 3 months, primary caregivers answered three questions, all on a 5-point Likert-type scale (never, rarely, sometimes, often, always):

- Have you used the Time Machine to budget your child’s television or computer use?
- How often did you use any of the strategies discussed in the monthly newsletters to modify your child’s television or computer use?
- In the last week, how often have you used any strategy to modify your child’s television or computer use?

At 6 months, primary caregivers completed a more detailed questionnaire consisting of the same three questions above, as well as further questions about the use of individual strategies, perceptions of their child’s behaviour change, and perceptions of the SWITCH intervention as a whole (for the wording of items, see Table 3). The response format was a 3- to 5-point Likert-type scales.

### Analysis

Data on intervention delivery (from community workers to primary caregivers) and intervention use (from primary caregiver to child) were presented descriptively. In addition, a post-hoc analysis was conducted to explore effects of level of primary caregiver implementation on child outcomes at 6 months, using the principle of intention to treat by using data collected from all randomised participants. Implementation was defined by primary caregiver response to the following question at 6 months: “In the last week, how often have you used any strategy to modify your child’s television or computer use?” with responses collapsed into four categories (never, rarely, sometimes or often/always). The adjusted regression model used in the main trial analysis [7] was used to evaluate the influence of primary caregiver use of the intervention on child BMI (kg/m^2^), zBMI, total sedentary behaviour (minutes/day) and screen-based sedentary behaviour (minutes/day) at 6 months. The new indicator replaced the treatment groups in this exploratory analysis to indicate the actual level of intervention implemented by primary caregiver during the trial, with all control participants allocated to the category of ‘never’ (i.e., reference group). All other covariates in the model remained the same (baseline outcome value, age, sex and ethnicity). As a sensitivity analysis, we used control participants only as the reference group, with the intervention responses collapsed into three categories (never/rarely, sometimes or often/always).

## Results

127 participants were randomly assigned to the intervention group, and 124 to the control group, with 121 (95 %) and 117 (94 %) respectively completing the six month assessment. Baseline characteristics of the sample are presented in Table 1, and results of the main trial analyses on primary and secondary outcomes are reported in Maddison et al. [7].

**Table 1:** Baseline characteristics of the sample (replicated in part from Maddison et al. [7])

	Intervention (*n* = 127)	Control (*n* = 124)
Age (years)	11.2	11.3
Gender		
Male	72 (57 %)	70 (56 %)
Female	55 (43 %)	54 (44 %)
Ethnicity		
Māori	16 (13 %)	13 (11 %)
Pacific	67 (53 %)	66 (53 %)
NZ/European	44 (34 %)	44 (35 %)
Refused to answer	0	1 (1 %)
Household income		
Under NZ$20,000	14 (11 %)	22 (18 %)
NZ$20,001–$30,000	14 (11 %)	21 (17 %)
NZ$30,001–$40,000	19 (15 %)	17 (14 %)
NZ$40,001–$50,000	18 (14 %)	14 (11 %)
NZ$50,001–$60,000	5 (4 %)	9 (7 %)
NZ$60,001–$70,000	11 (9 %)	4 (3 %)
NZ$70,001–$80,000	9 (7 %)	7 (6 %)
NZ$80,001–$90,000	6 (5 %)	6 (5 %)
Over NZ$90,000	19 (15 %)	12 (10 %)
Don’t know	9 (7 %)	12 (10 %)
Refused to answer	3 (3 %)	0 (0 %)

### Intervention delivery

Intervention monitoring was completed for three out of four SWITCH community workers (one changed employment before monitoring could be completed). The monitoring indicated that community workers delivered all core intervention components to primary caregivers.

A common thread of the monitored meetings was time restraints imposed by the primary caregiver, for example the immediate needs of other young children in the house. This resulted in the community workers delivering a lower level of detail on some of the strategies than intended, to ensure all strategies were covered in the time available. Additionally, in one of the monitored visits, the Time Machine was incompatible with the primary caregiver’s television due to an HDMI cable. In these cases, community workers left the device with the family. The device could then still be used as a timer, though it would not switch off the television automatically.

### Intervention use/uptake

Self-reported use of the intervention at 3 and 6 months is presented in Tables 2 and 3, respectively. A similar pattern of response was found at both time points, albeit with a higher proportion of missing data at 6 months.

**Table 2:** Caregiver self-reported use of intervention at 3 months (*n* = 127)

Question	Number	Percent
Have you used the Time Machine to budget your child’s television or computer use?		
Never	76	59.8
Rarely	16	12.6
Sometimes	12	9.5
Often	3	2.4
Always	9	7.1
Missing	11	8.7
How often did you use any of the strategies discussed in the monthly newsletters to modify your child’s television or computer use?		
Never	15	11.8
Rarely	24	18.9
Sometimes	44	34.7
Often	27	21.3
Always	6	4.7
Missing	11	8.7
In the last week, how often have you used any strategy to modify your child’s television or computer use?		
Never	22	17.3
Rarely	29	22.8
Sometimes	29	22.8
Often	26	20.5
Always	10	7.9
Missing	11	8.7

**Table 3:** Caregiver self-reported use of intervention at 6 months (*n* = 127)

Question	Number	Percent
Have you used the Time Machine to budget your child’s television or computer use?		
Never	58	45.7
Rarely	18	14.2
Sometimes	14	11.0
Often	8	6.3
Always	2	1.6
Missing	27	21.3
Were you able to stick to your child’s screen time budget?		
Never	40	31.5
Rarely	7	5.5
Sometimes	28	22.1
Often	22	17.3
Always	3	2.4
Missing	27	21.3
Did you add more time to the Time Machine before the week was up?		
No, never	68	53.5
Yes, a couple of times	18	14.2
Yes, some of the time	10	7.9
Yes, most of the time	2	1.6
Yes, all of the time	2	1.6
Missing	27	21.3
Do you think using a Time Machine is a good way to reduce children’s screen time?		
Yes	47	37.0
No	16	12.6
Not applicable	44	34.7
Missing	20	15.8
How often did you use any of the strategies discussed in the monthly newsletters to modify your child’s television or computer use?		
Never	23	18.1
Rarely	12	9.5
Sometimes	43	33.9
Often	24	18.9
Always	5	3.9
Missing	20	15.8
In the last week, how often have you used any strategy to modify your child’s television or computer use?		
Never	27	21.3
Rarely	25	19.7
Sometimes	25	19.7
Often	20	15.8
Always	10	7.9
Missing	20	15.8
Did the amount of time your child spent on screen activity decrease over the 6 months?		
Yes	95	74.8
No	12	9.5
Missing	20	15.8
If Yes (*n* = 95), what activity did your child most frequently replace screen time with?		
A physical activity inside the house	6	6.3
A physical activity outside	57	60.0
Another sitting activity (such as reading, listening to music, board games)	32	33.7
The length of this programme was 6 months. What do you think about this?		
Too long	19	15.0
Just the right amount of time	80	63.0
Not long enough	8	6.3
Missing	20	15.8

Most primary caregivers did not use the Time Machine to budget television/video game time; however, one third reported that the Time Machine was a good way to reduce children’s screen-based sedentary behaviour at 6 months. Approximately two thirds of primary caregivers reported using any of the intervention strategies presented in either the face-to-face meeting or the subsequent newsletters “sometimes” or less at both time points. However at 6 months, three quarters of primary caregivers felt that their child’s screen time had reduced and the reduced time was predominantly replaced by outdoor physical activity.

Use of individual intervention strategies at 6 months is presented in Table 4. The most frequently used strategies were praise and positive reinforcement, rule setting, and encouraging alternative activities. However, the post-hoc exploratory analysis indicated no significant effect of primary caregiver use of the intervention on body composition or sedentary behaviour in children (Table 5). The sensitivity analysis also found no significant effect of intervention use on outcomes.

**Table 4:** Caregiver self-reported use of individual intervention strategies at 6 months (*n* = 127)

Question	Number	Percent
Did you use any of the following parts of the programme?		
Praise and positive reinforcement (face-to-face meeting)		
Never	6	4.7
Rarely	8	6.3
Sometimes	24	18.9
Often	53	41.7
Always	16	12.6
Missing	20	15.8
Re-arranging the home environment (face-to-face meeting)		
Never	29	22.8
Rarely	15	11.8
Sometimes	34	26.8
Often	19	15.0
Always	10	7.9
Missing	20	15.8
TV time budgeting (face-to-face meeting)		
Never	25	19.7
Rarely	9	7.1
Sometimes	37	29.1
Often	27	21.3
Always	9	7.1
Missing	20	15.8
Encouraging alternative activities (face-to-face meeting)		
Never	5	3.9
Rarely	3	2.4
Sometimes	25	19.7
Often	49	38.6
Always	25	19.7
Missing	20	15.8
Family support and role modelling (face-to-face meeting)		
Never	7	5.5
Rarely	10	7.9
Sometimes	38	29.9
Often	37	29.1
Always	15	11.8
Missing	20	15.8
Contingency management (newsletter)		
Never	13	10.2
Rarely	16	12.6
Sometimes	42	33.1
Often	27	21.3
Always	9	7.1
Missing	20	15.8
Shaping the desired behaviour in gradual steps (newsletter)		
Never	10	7.9
Rarely	12	9.5
Sometimes	41	32.3
Often	29	22.8
Always	15	11.8
Missing	20	15.8
Setting rules for TV/computer use (newsletter)		
Never	8	6.3
Rarely	2	1.6
Sometimes	24	18.9
Often	44	34.7
Always	29	22.8
Missing	20	15.8
Switch off challenges (newsletter)		
Never	25	19.7
Rarely	23	18.1
Sometimes	32	25.2
Often	18	14.2
Always	9	7.1
Missing	20	15.8

**Table 5:** Child outcomes by caregiver implementation at 6 months (*n* = 231)^a^

	Number	BMI (kg/m^2^)		zBMI		SSB (min/day)		TSB (min/day)	
		Model-adjusted mean (95 % CI)	*P* value*	Model-adjusted mean (95 % CI)	*P* value*	Model-adjusted mean (95 % CI)	*P* value*	Model-adjusted mean (95 % CI)	*P* value*
Implementation									
Often/always	30	26.79 (26.32 to 27.26)	0.37	2.60 (2.51 to 2.69)	0.33	200 (145 to 255)	0.43	541 (491 to 591)	0.81
Sometimes	25	27.09 (26.58 to 27.60)	0.06	2.64 (2.54 to 2.74)	0.11	219 (159 to 280)	0.88	544 (490 to 599)	0.73
Rarely	25	26.32 (25.81 to 26.83)	0.40	2.47 (2.37 to 2.57)	0.16	204 (142 to 266)	0.55	508 (451 to 564)	0.40
Never	151	26.56 (26.34 to 26.77)	ref	2.55 (2.51 to 2.59)	ref	225 (199 to 251)	ref	534 (510 to 558)	ref

*BMI* body mass index, *CI* confidence interval, *kg* kilograms, *m* metres, *min* minutes, *ref* reference group, *SSB* screen-based sedentary behaviour, *TSB* total sedentary behaviour, *zBMI* body mass index z-score

*P value indicates statistical significance in mean difference between the current and reference level of caregiver implementation

^a^Twenty intervention primary caregivers did not provide information on implementation. Regression analysis controlled for baseline outcome value, age, sex and ethnicity

## Discussion

Process evaluations may be used to shed light on how interventions work [1, 2]. In this process evaluation, we chose to focus on intervention implementation. Three levels of implementation (from investigative team to SWITCH community worker, from community worker to primary caregiver, and from primary caregiver to child) were employed to deliver a number of screen time reduction strategies, which could be combined and delivered as the primary caregiver saw fit. The SWITCH intervention was designed to be pragmatic and scalable, and was therefore less intensive than previous similar interventions in children [12–15].

Consistent with a recent systematic review of screen time interventions in children [16], the SWITCH study found no significant intervention effect on health behaviours or body composition. This may be related to the low intensity of the intervention; however, the results of this process evaluation suggest that the uptake of the intervention by primary caregivers was low, and that the strategies the primary caregivers chose to implement lacked efficacy, which likely compounded this issue. The SWITCH intervention was delivered by community workers in a broadly standardised way, but the monitoring highlighted the issue of competing responsibilities for the primary caregiver and the subsequent difficulty in delivering all of the intended detail. Additionally, of the ten strategies delivered in the intervention, only four (praise and positive reinforcement, rule setting, and encouraging alternative activities) appeared to be well-used by primary caregivers. These four strategies may be viewed as the intervention’s “active ingredients” in that of the suite of options available, these were the ones implemented at least to some degree, though they still did not impact on behaviour. It was noteworthy that high engagement with study itself (reflected by the low attrition) did not appear to correspond to high engagement with the intervention.

It remains speculative as to whether the intervention would have been effective if other, more, or all of the evidence-based strategies had been used by primary caregivers. For example, the home screen environment has been shown to be an important determinant of screen use in children [17], yet the majority of primary caregivers did not report using the environmental control strategy (i.e., re-arranging the home environment to make it less conducive to screen time). Despite television locking devices being a promising avenue in recent reviews [18], and being well-used - albeit with moderate acceptability - in a pilot study conducted prior to the main SWITCH trial [19] the majority of primary caregivers never used the Time Machine in this study. The lack of popularity of the Time Machine is at odds with the finding that one third of primary caregivers thought these devices were a useful screen time reduction strategy. This discrepancy may be the result of social desirability bias, or the feeling that the devices were useful generally, but not in their family specifically. Finally, the compatibility issues between the Time Machine and newer television technologies (e.g., HMDI cables), and more broadly the development of personal mobile screens, which make controlling the home screen environment more difficult, highlight the pitfalls of conducting an intervention with a technological component when technology advances so rapidly.

Despite the poor intervention uptake and efficacy suggested by the process evaluation, most primary caregivers reported that their child’s screen time had reduced and this time was replaced by physical activity, though again social desirability bias may have been a factor. This finding corroborates somewhat with the secondary outcomes of the trial, which indicated (non-significant) decreases in child-reported screen-based sedentary time (−33.2 min/day, 95 % CI −73.3 to 7.0, *p* = 0.11) and commensurate increases in child-reported moderate intensity physical activity (24.3 min/day, 95 % CI −0.9 to 49.5, *p* = 0.06) [7].

The SWITCH intervention was broadly based on a previous intervention by Epstein and colleagues in the United States (US) [12]. This was a 2-year family intervention to reduce screen-based sedentary behaviour in children aged 4–7 years. At 2 years, screen time (−17.5 h per week vs. -5.2 h per week, *p* < 0.001) and zBMI (−0.24 units vs. -0.13 units, *p* < 0.05) declined by a greater extent in the intervention group than control. In the SWITCH intervention, modifications were made to incorporate the local NZ sociocultural context, financial incentives to reduce children’s screen time were not used because of concerns about sustainability, and the intervention was designed to be pragmatic (i.e., able to be rolled out on a national level) and was thus much less intensive and shorter duration than the Epstein intervention. Use of the television budgeting device was compulsory and formed the cornerstone of the intervention in the Epstein trial, but was not compulsory (and the devices not well used) in the present study.

While the content was similar, the Epstein trial may be seen as a predominantly environmental intervention supplemented by a behavioural component and vice versa for the SWITCH trial. The SWITCH intervention was not a direct replication of the Epstein intervention, and the modifications (though carefully reasoned) may have changed the fundamental nature of the intervention, in particular the decision not to use financial incentives. The lowered “dose” of the intervention in SWITCH, coupled with poor uptake, likely further contributed to the lack of success of the SWITCH intervention. The success of the intervention in the US did not appear to generalise to a NZ context. Despite an extended process of consultation and subsequent modifications to incorporate sociocultural factors unique to NZ, as well as high Maori (indigenous population) and Pacific (ethnic minority) participation (12 and 53 % of the study population, respectively), the poor uptake and efficacy of the SWITCH intervention may be related to a lack of acceptability of some components. The Epstein trial used this intervention in a considerably younger group of children than the SWITCH trial (4–7 years versus 9–12 years). This type of family-based, primary caregiver-administered intervention may be more efficacious in younger children where parental influence is stronger [20].

The strengths of this process evaluation include using a pre-planned approach to examine the implementation of the intervention at multiple levels, and at both mid- and end-trial. The strengths of the SWITCH study overall include a randomised design, use of an evidence- and theory-based intervention, adequate sample size to detect change on the primary outcome and a high proportion of Maori and Pacific participation [7]. A key limitation of the process evaluation is that for pragmatic reasons, community workers were monitored only once during a face-to-face meeting. Therefore, the monitoring gave an indication of the consistency with which intervention components were delivered, and a flavour of the issues experienced during fieldwork, but was not a comprehensive evaluation of intervention delivery. Additionally, intervention use by primary caregivers was assessed using a limited number of fixed-response items. Finally, the post-hoc analysis of the effect of primary caregiver implementation on child outcomes was likely underpowered and was exploratory/hypothesis generating only; thus, it should be interpreted with caution. Overall, this process evaluation gave an indication of *how* the intervention was implemented, and whether the strategies implemented appeared to be efficacious, but it failed to adequately address the overarching question of *why* some components were used or useful (or not). In future trials, this may be most usefully assessed using a qualitative approach.

These findings have implications for the design of future interventions. Given the difficulties encountered delivering all intervention content in the face-to-face meetings, multiple, shorter sessions with the caregiver, or supplementary telephone sessions, may be considered for future research. While resource- and time-intensive, an extended iterative process of intervention development may be useful both prior to, and perhaps during, the trial to enhance the efficacy of intervention components. In this type of complex intervention, implementation is likely to be an issue, so procedures to regularly monitor and bolster implementation should be built into the study design from the outset. For example, this could include additional phone or email contacts aimed at sustaining motivation and problem-solving implementation issues. It may be that the implementation chain (investigative team to community worker to primary caregiver to child) was simply too long in the SWITCH trial, and future family interventions may benefit from an additional component directly targeting the child. One final lesson is to think carefully about tailoring the intervention in light of contextual issues and characteristics of the participants (even if the intervention has been efficacious in other populations or studies) to optimise the effects of the intervention on the target behaviour.

## Conclusions

In conclusion, this process evaluation of the SWITCH trial indicated that poor uptake of intervention components, and weak efficacy of the intervention itself, may have played a role in the null findings on health behaviour and body composition.

## Abbreviations

ANCOVA, analysis of covariance; BMI, body mass index; HDMI, high-definition multimedia interface; NZ, New Zealand; SWITCH, The Screen Time Weight-loss Intervention Targeting Children at Home trial; US, United States of America; zBMI, body mass index z-score
